# Expansion of different subpopulations of CD26^−/low^ T cells in allergic and non-allergic asthmatics

**DOI:** 10.1038/s41598-019-43622-8

**Published:** 2019-05-17

**Authors:** Juan José Nieto-Fontarigo, Francisco Javier Salgado, María Esther San-José, María Jesús Cruz, Luis Valdés, Amparo Pérez-Díaz, Pilar Arias, Montserrat Nogueira, Francisco Javier González-Barcala

**Affiliations:** 1 Department of Biochemistry and Molecular Biology, Faculty of Biology-Biological Research Centre (CIBUS), Universidade de Santiago de Compostela, Santiago de Compostela, Spain; 2 Clinical Analysis Service, University Hospital of Santiago de Compostela, Santiago de Compostela, Spain; 3 Department of Respiratory Medicine-Hospital Vall d’Hebron, Universitat Autònoma de Barcelona, Barcelona, Spain; 4 Spanish Biomedical Research Networking Centre-CIBERES, Barcelona, Spain; 5 Department of Medicine, Universidade de Santiago de Compostela, Santiago de Compostela, Spain; 6 Department of Respiratory Medicine, University Hospital of Santiago de Compostela, Santiago de Compostela, Spain; 7 Health Research Institute of Santiago de Compostela (IDIS), Santiago de Compostela, Spain; 8 Drug Screening Platform/Biopharma Research Group, Molecular Medicine and Chronic Diseases Research Centre (CIMUS), Universidade de Santiago de Compostela, Santiago de Compostela, Spain

**Keywords:** Translational immunology, Asthma, T cells

## Abstract

CD26 displays variable levels between effector (TH_17_ ≫ TH_1_ > TH_2_ > Treg) and naïve/memory (memory > naïve) CD4^+^ T lymphocytes. Besides, IL-6/IL^−^6R is associated with TH_17_-differentiation and asthma severity. Allergic/atopic asthma (AA) is dominated by TH_2_ responses, while TH_17_ immunity might either modulate the TH_2_-dependent inflammation in AA or be an important mechanism boosting non-allergic asthma (NAA). Therefore, in this work we have compared the expression of CD26 and CD126 (IL-6Rα) in lymphocytes from different groups of donors: allergic (AA) and non-allergic (NAA) asthma, rhinitis, and healthy subjects. For this purpose, flow cytometry, haematological/biochemical, and *in vitro* proliferation assays were performed. Our results show a strong CD26-CD126 correlation and an over-representation of CD26^−^ subsets with a highly-differentiated effector phenotype in AA (CD4^+^CD26^−/low^ T cells) and NAA (CD4^−^CD26^−^ γδ-T cells). In addition, we found that circulating levels of CD26 (sCD26) were reduced in both AA and NAA, while loss of CD126 expression on different leukocytes correlated with higher disease severity. Finally, selective inhibition of CD26-mRNA translation led to enhanced T cell proliferation *in vitro*. These findings support that CD26 down-modulation could play a role in facilitating the expansion of highly-differentiated effector T cell subsets in asthma.

## Introduction

Asthma is a heterogeneous disease with different phenotypes (e.g. allergic asthma/AA and non-allergic asthma/NAA) and endotypes that remain poorly understood^1–4^. The major endotype classification of asthma is based on the predominant T helper (TH)-type inflammation^5^. On the one hand, TH_2_^high^ asthma is the most common endotype, characterised by an eosinophilic and TH_2_-driven inflammation and a central role for IgE^1,6^. On the other hand, TH_2_^low^ asthma is more heterogeneous, including neutrophilic and paucygranulocytic inflammation and an involvement of TH_1_ and TH_17_ cells^1,6^. TH_17_ cells have been associated with asthma severity^7,8^. Indeed, IL-17 has been implicated in steroid resistance, airway remodeling, and the induction of neutrophilic inflammation^9,10^.

In 2012, Bengsch *et al*. highlighted the use of CD26 as a marker for human TH_17_ cells^11^. Moreover, they showed that the expression of CD26 amongst Teff cells is variable (TH17 ≫ TH1 > TH2)^11^, while our group described this molecule as a negative selection marker for regulatory T cells (Treg cells)^12^. CD26/Dipeptidyl peptidase 4 (DPP4) is a “moonlighting” protein: it is an enzyme that inactivates important soluble factors (e.g. cytokines like IL-3; chemokines like CCL11/Eotaxin or CCL5/RANTES; incretins) and also a protein with proteolytic-independent roles (e.g. co-stimulation)^13–15^. Furthermore, CD26^high^ TH lymphocytes are considered memory^16,17^ or activated cells^18^, which explains the presence of CD26^high^CD4^+^ T cells in AA^19,20^. A soluble version of CD26 (sCD26) has been found in the bloodstream as a free or a vesicle-associated protein [http://www.exocarta.org]^21^. Our previous *in vitro* studies evidenced a positive correlation between soluble DPP4 activity (an indirect measurement of sCD26) and CD26 expression on CD4^+^ cells^20^. Immune cells also appear to be a source of sCD26 *in vivo*^15^. However, contrary to our expectations, allergic asthmatics displayed higher membrane expression of CD26 on CD4^+^ T cells, but decreased levels of sCD26. This finding can be explained through the expansion of a “triple low” (Tlow; CD25^−^CD127^−^CD26^−/low^) subpopulation of effector T cells (Teff)^20^.

IL-6 is an important cytokine in the differentiation of TH_17_ cells that acts via IL-6R (IL-6Rα/CD126 + gp130)^22^. In this sense, this cytokine down-modulates the TGF-β-driven expression of FoxP3 and up-regulates the levels of the transcription factor that controls the development of Th17 cells: RORγt^23,24^. IL-6 signalling is also essential for the generation of functionally active memory CD4^+^ T cells^25^. Like CD26, CD126 is also found in plasma as a soluble molecule: sIL-6Rα. This circulating protein binds to IL-6 and leads to the activation of CD126^−^gp130^+^ cells, a process known as *trans*-signalling^26,27^. Indeed, CD4^+^ T cells down-modulate IL-6R upon inflammatory activation, but these cells retain the IL-6 response capacity through the *trans*-signalling pathway^28^, a mechanism of paramount importance for the maintenance of inflammatory diseases such as asthma^29–32^.

Most research in asthma has been focused on the allergic phenotype and CD4^+^ T cells. However, CD4^−^ lymphocyte subsets (e.g. CD8^+^ αβ-T cells or γδ-T lymphocytes) might also be relevant for this disease and its phenotypes/endotypes. Thus, γδ-T lymphocytes become activated by IL-6 *trans-*signalling^33^, and they are important inducers of allergic asthmatic responses^34^. Moreover, they are major initial producers of IL-17^35^. On the other hand, CD8^+^ T cells cooperate with CD4^+^ T cells to promote asthma and have been associated with poor lung fuction and airway obstruction^36–39^. Interestingly, the expression of both CD26 and CD126 defines diverse stages of differentiation of CD8^+^ T cells^40,41^, which might be modified in asthma^41^. Given this differential expression of CD26 and CD126 between different lymphocyte subsets, we postulate that both molecules might display coordinated expression levels and help distinguish different asthma phenotypes. Therefore, the aim of our study was to analyse the expression of CD26 and CD126 in CD4^+^ and CD4^−^ lymphocytes from healthy subjects and patients with AA/NAA or rhinitis.

Herein, we report a high correlation between the expression of CD126 and CD26 in lymphocytes. These molecules help differentiate between lymphocytes with naïve (CD26^intermediate^ or CD26^int^), central-memory (CD26^high^), or “terminally-differentiated” (T_EMRA_)/effector-memory (T_EM_) (CD26^−/low^) phenotypes. Moreover, allergic and non-allergic asthmatics display low circulating sCD26 levels, which is consistent with the expansion of CD26^−/low^ T_EM_/T_EMRA_ lymphocytes: CD4^+^CD26^−/low^ T cells (previously named Tlow cells)^20^ in AA and CD4^−^CD26^−^ γδ-T cells in NAA. Acceleration of the natural course of CD26 down-modulation on T lymphocytes by siRNA leads to higher *in vitro* proliferation rates, which suggests that CD26 molecules on T lymphocytes could be acting as a “brake mechanism” that prevents their proliferation and the acquisition of an effector phenotype. Finally, a decrease in the number of CD126 molecules on leukocytes correlates with higher asthma severity. Thus, our findings provide new advances in asthma immunophenotyping and on the role of CD26/CD126 in this disease.

## Results

### Characteristics of study subjects

We performed a case-control study including adult patients with different asthma phenotypes (AA; NAA), rhinitis and healthy controls (HC). The characteristics of the donors in this study are summarized in Table 1. Pulmonary function parameters (FEV1 and FEV1/FVC) were lower in both AA and NAA relative to patients with rhinitis (Table 1). Haematological count revealed an increment of eosinophil numbers in asthma patients (both AA and NAA) compared to HC (Table 1). Furthermore, AA displayed higher blood eosinophil counts than patients with rhinitis (Table 1). Levels of other leukocyte populations remained unchanged (Table 1).

**Table 1:** Characteristics of the study population.

	AA	NAA	R	HC
**N**	100	92	44	32
Age (mean (range))	36 (18–68)	52 (22–72)	35 (18–55)	43 (22–61)
Sex (M/F)	46/54	22/70	25/19	15/17
**Disease control**				
Yes	83	75	44	—
No	17	17	0	—
**Baseline treatment**				
ICS-LABA	79	75	0	0
ICS	16	4	0	0
OCS	1	0	0	0
Antileukotrienes	41	31	11	0
Anticholinergic	15	28	0	0
Roflumilast	0	2	0	0
Prednisone	0	5	0	0
Biochemical, hematological and pulmonary function parameter				
FEV1 (%)	97.0 (88.3–107.0)^#^	97.0 (73.2–112.0)^#^	108.0 (99.0–119.0)	—
FEV1/FVC (%)	76.0 (70.5–80.4)^#^	73.7 (66.2–80.0)^#^	83.4 (79.0–87.2)	—
Neutrophils (10^3^ cells/μL)	3.70 (3.03–4.29)	3.56 (3.08–4.30)	3.56 (2.89–3.99)	3.02 (2.28–4.05)
Lymphocytes (10^3^ cells/μL)	1.99 (1.67–2.29)	1.94 (1.58–2.34)	2.14 (1.78–2.58)	1.99 (1.53–2.49)
Monocytes (10^3^ cells/μL)	0.40 (0.31–0.47)	0.37 (0.30–0.51)	0.39 (0.31–0.46)	0.39 (0.33–0.49)
Eosinophils (10^3^ cells/μL)	0.29 (0.20–0.47)^&#^	0.28 (0.16–0.41)^&^	0.22 (0.14–0.29)	0.13 (0.09–0.22)
Basophils (10^3^ cells/μL)	0.04 (0.03–0.05)	0.03 (0.02–0.05)	0.04 (0.02–0.05)	0.03 (0.02–0.05)
ESR (1 h; mm)	7.5 (4.0–15.0)*	12.0 (7.0–20.8)^#&^	8.0 (2.0–14.7)	7.5 (2.0–10.0)
TGF-β1 (ng/mL)	14751 (11882–17299)	13712 (10118–18149)	13957 (8691–19056)	15654 (11552–18620)
sCD25 (ng/mL)	2.86 (2.29–3.64)	3.02 (2.42–3.90)	2.77 (2.29–4.09)	2.77 (2.3–4.13)
IgE (IU/mL)	167.5 (72.0–301.0)*^#&^	28.0 (10.0–60.5)^#^	66.0 (24.0–115.0)	15.0 (5.7–55.7)
IgG (mg/dL)	1075 (943–1200)	1035 (898–1160)	1010 (913–1200)	—
IgG1 (mg/dL)	659 (563–745)*	573 (461–677)	617 (530–743)	—
IgG2 (mg/dL)	346 (337–429)	337 (263–429)	315 (259–411)	—
IgG3 (mg/dL)	33 (23–49)	37 (28–56)	34 (28–48)	—
IgG4 (mg/dL)	48 (27–85)^#^	40 (19–67)	34 (21–51)	—
IgA (mg/dL)	226 (167–295)	213 (159–263)	191 (133–274)	—
IgM (mg/dL)	98.5 (72–147)	113 (75–151)	111 (69–166)	—
TNF (pg/mL)	6.8 (5.5–8.5)	6.2 (5.4–7.6)	6.8 (5.2–9.1)	—

AA, allergic asthmatics; HC, healthy controls; NAA, non-allergic asthmatics; R, rhinitis patients.

Data are presented as median value (IQR1–3), unless otherwise expressed.

Kruskal–Wallis test followed by Dunn’s multiple comparison test (P < 0.05). *AA vs NAA; ^#^asthma vs R; ^&^asthma vs HC.

As expected, AA subjects exhibited higher levels of IgE than other study groups (Table 1), and the same happened for rhinitis compared to NAA (Table 1). Moreover, our results evidenced a reduction of IgG1 in NAA relative to AA, while IgG4 was higher in AA *vs*. R (Table 1). We also analysed the erythrocyte sedimentation rate (ESR), which was elevated in NAA *vs*. remaining groups (Table 1).

### CD26 and CD126 molecules display a highly correlated expression on lymphocytes, while the expansion of “triple low” (CD25^−^CD26^−^CD127^−^) CD4^+^ T cells explains the reduction of sCD26 in allergic asthma

**Figure 1:**
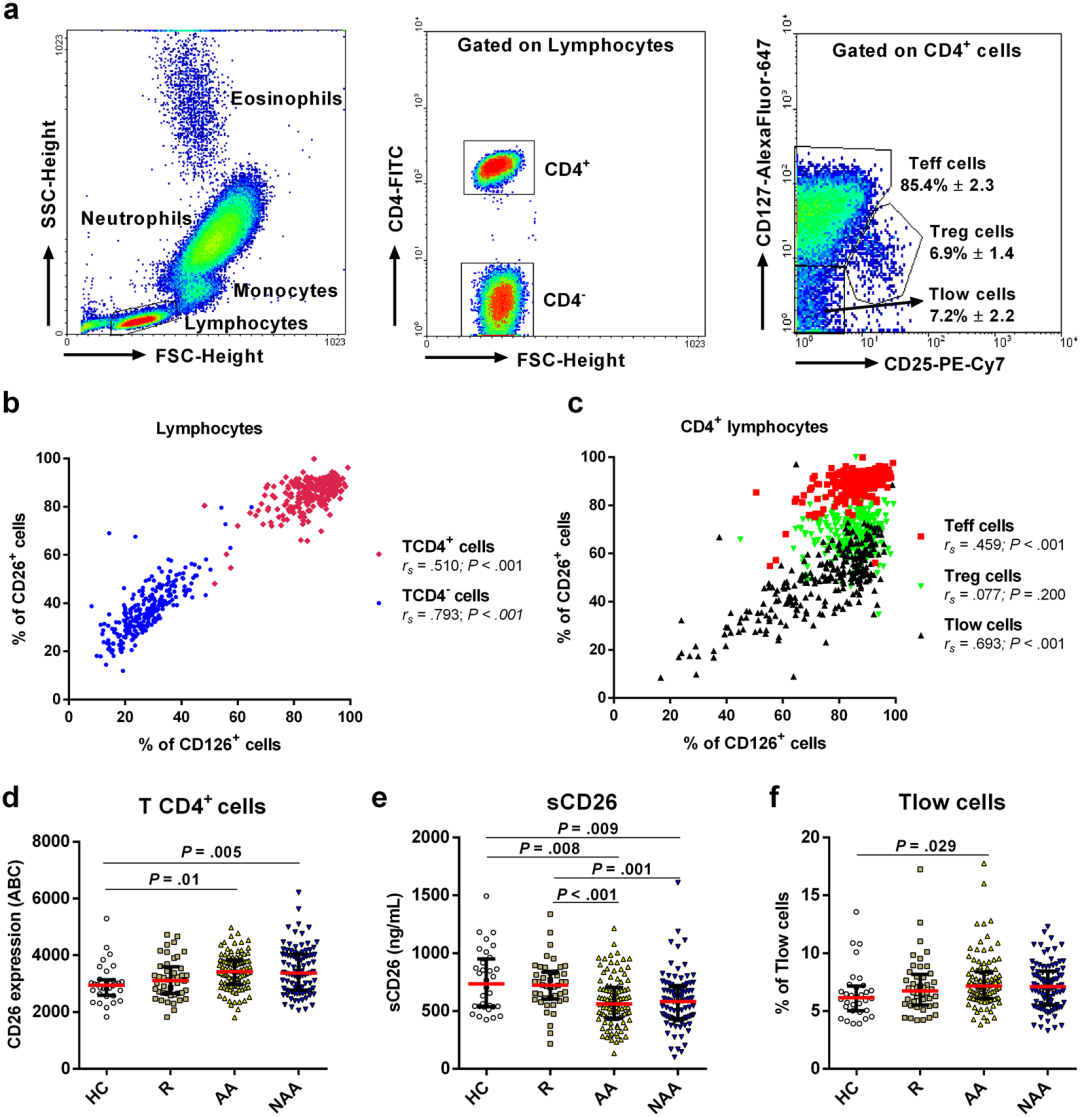
CD26 and CD126/IL-6Rα display a coordinated expression on effector CD4^+^ T cells, but only CD26/sCD26 levels are altered in asthma. (**a**) Gating strategy in flow cytometry assays to identify relevant CD4^+^ T cell subsets. Lymphocytes were gated based on the forward (FSC-Height) and side-scatter (SSC-Height) parameters (left dot plot). Then, TH lymphocytes were selected according to their high CD4 expression (middle dot plot). Finally, levels of CD25 and CD127 were used to identify three different TH subsets (right dot plot): “conventional” Teff cells (Teff), regulatory T cells (Treg), and “triple low” Teff cells (Tlow). (**b**,**c**) Correlation between the expression (% of positive cells) of CD26 and CD126 on CD4^+^ (red) or CD4^−^ (blue) lymphocytes (**b**) or within different CD4^+^ subsets (**c**): “conventional” Teff (red), Treg (green), and Tlow cells (black) (data from all study subjects; N = 268). (**d**) Expression of CD26 (median ± IQR1-3) presented as number of antibody molecules bound per cell (ABC; see Material and Methods section) in healthy controls (HC; N = 32), rhinitis (R; N = 44), allergic asthma (AA; N = 100), and non-allergic asthma (NAA; N = 92). (**e**) Serum levels of sCD26 (ng/mL) measured by ELISA. (**f**) Percentage of Tlow cells within the CD4^+^ T cell gate. (**d**–**f**) Statistically significant differences between groups are indicated (Kruskal-Wallis test: *P* < 0.05).

T cells account for ~80% circulating lymphocytes and include CD4^−^ and CD4^+^ cells. CD4^+^ lymphocytes (TH) were subdivided into “conventional” Teff (CD25^low^CD127^high^), Treg (CD25^high^CD127^low^), and Tlow cells (CD25^low^CD127^low^) (Fig. 1a). TH cells displayed the highest percentage of CD26^+^ cells within leukocytes (~85–90%), and this parameter evidenced a positive association with the percentage of CD126^+^ cells (Fig. 1b). Amongst TH lymphocytes, “conventional” Teff (CD26^+/high^) and especially Tlow (CD26^−/low^) cells exhibited the same high CD26-CD126 correlation, but Tregs appear to lose this association (Fig. 1c). Furthermore, we found a reinforced expression of CD26 (measured in Antibody Bound per Cell/ABC; see Material and Methods section) on CD4^+^ T cells from AA and NAA patients (Fig. 1d) compared to HC. Strikingly, CD126 showed an unaltered expression in CD4^+^ T cells from the different groups of donors (data not shown) despite the strong correlation between CD26 and CD126 commented above (Fig. 1b).

The higher number of CD26 molecules on CD4^+^ T cells from AA and NAA patients compared to HC (Fig. 1d) was in line with the expected activated phenotype of these cells, but contrasted with the decreased levels of sCD26 in asthmatics (Fig. 1e). However, as most of sCD26 comes from T cells^42^, the expansion of CD26^−/low^ T lymphocyte populations may explain the decreased levels of sCD26 in AA. Indeed, even though Teff and Treg proportions remained unchanged, we detected an expanded subpopulation of CD25^−^CD127^−^CD26^−/low^ (Tlow) effector T cells in AA patients compared to HC (Fig. 1f). Moreover, contrary to CD4^+^ and “conventional” Teff cells (CD26^int/high^ subsets) and despite the CD26^−/low^ phenotype of these Tlow cells, the percentage of the last subset was negatively correlated with the percentage of CD26^+^ and CD126^+^ cells within the Tlow compartment (Supplementary Table S1). Interestingly, the percentage of CD127^+^ Treg cells was augmented in asthmatics compared to rhinitis and HC (Supplementary Fig. S1).

### The CD4^+^CD26^−/low^ T compartment from allergic asthmatics includes memory cells in an advanced stage of differentiation

**Figure 2:**
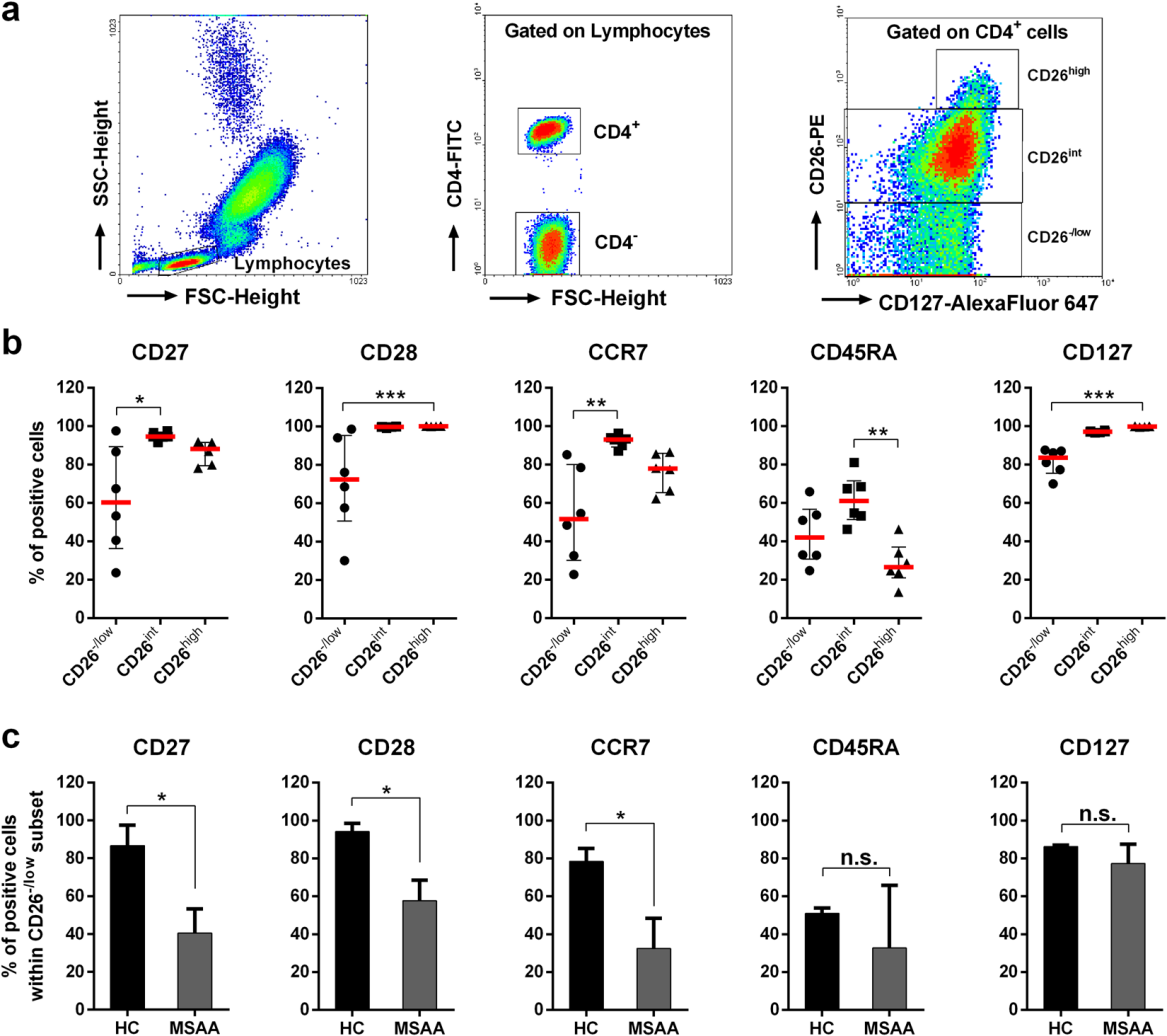
CD26^−/low^ CD4^+^ T cells contain effector lymphocytes with an advanced differentiation stage. (**a**) CD4^+^ T cells were identified by using the same strategy as in Fig. 1a. Then, CD127 and CD26 markers were used to delimitate three CD4^+^ lymphocyte subsets (right dot plot): CD26^−/low^ (including CD127^−^ and CD127^‘+^ cells), CD26^int^ and CD26^high^. (**b**) Phenotyping of CD26^−/low^, CD26^int^, and CD26^high^ subsets according to the surface expression of CD27, CD28, CCR7, CD45RA, and CD127. Data were obtained from 6 donors (3 healthy subjects and 3 moderate-severe allergic asthmatics) and expressed as % of positive cells for each marker (median ± IQR1-3). Kruskal-Wallis test followed by Dunn’s multiple comparison post-hoc analysis was used to assess significant changes between groups. *P < 0.05, **P < 0.01, ***P < 0.001. (**c**) Expression of each marker (Percentage of positive cells; median ± IQR1-3) in CD26^−/low^ lymphocytes between healthy subjects (HC; N = 3) and moderate-severe allergic asthmatics (MSAA; N = 3). *t-test* was used to assess significant changes between HC and MSAA. *P < 0.05.

CD26 and CD127 are useful markers for identifying effector/memory T cells^11,16,17^. Based on these molecules, CD4^+^ lymphocytes were segregated in CD26^−/low^, CD26^int^, and CD26^high^ cells (Fig. 2a). As Fig. 2b illustrates, CD26^int^ and CD26^high^ populations showed elevated expression of co-stimulatory (CD27, CD28) and lymph node homing (CCR7) molecules, indicating an early differentiation stage. In addition, the CD26^high^ subpopulation was mainly composed of CD45RA^−^ cells (i.e. central-memory T cells/T_CM_; CD45RA^−^CCR7^+^CD27^+^CD28^+^), whereas most of naïve T lymphocytes (CD45RA^+^CCR7^+^CD27^+^CD28^+^) were included within the CD26^int^ subset (Fig. 2b). In contrast, CD26^−/low^ lymphocytes showed intermediate levels of CD45RA and a decreased expression of CD27, CD28, CCR7, and CD127 molecules (Fig. 2b), which agrees with an advanced differentiation stage (likely T_EM_ or T_EMRA_, CD45RA^+^CCR7^−^CD27^−^CD28^−^). Moreover, down-modulation of CD27, CD28, and CCR7 was mainly observed in CD26^−/low^ cells from patients with moderate-severe AA (Fig. 2c), the group of donors where Tlow cells (CD4^+^CD25^−^CD127^−^CD26^−/low^) were found expanded (Fig. 1f). Apart from Tlow cells, the CD26^−/low^ compartment within the TH subset is also occupied by Treg lymphocytes (CD25^high^CD127^low^; Supplementary Fig. S2), as Bailey *et al*. have previously reported^17^. However, 57.5 ± 7.3% of Treg lymphocytes are also found within the CD26^int^ subset (Supplementary Fig. S2). In summary, our results evidenced that: (a) most of naïve CD4^+^ T lymphocytes display intermediate expression levels of CD26; (b) The CD26^high^ subpopulation is mainly composed of T_CM_ cells; and (c) Tlow cells expanded in AA are likely effector T lymphocytes that have lost CD26 expression to become T_EM_ or T_EMRA_ cells (CD27^−^CD28^−^CCR7^−^CD45RA^+/−^).

### Non-allergic asthmatics display increased proportions of CD26^−^CD127^−^ and CD126^−^CD127^−^ subpopulations amongst CD4^−^ lymphocytes compared to allergic patients

**Figure 3:**
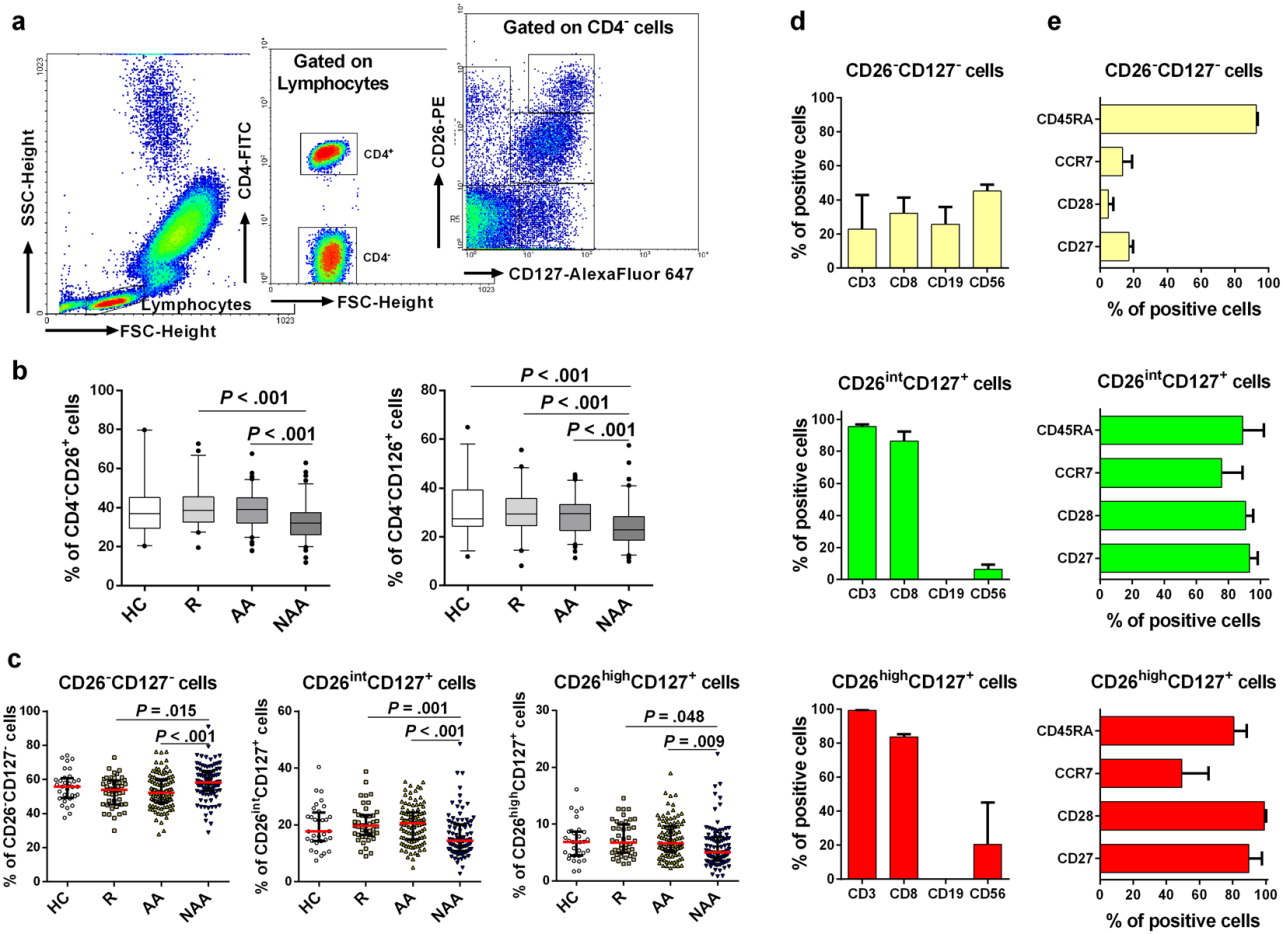
CD4^−^ lymphocytes with a highly differentiated memory phenotype are expanded in non-allergic asthma. (**a**) CD4^−^ lymphocytes were identified by flow cytometry (left and middle dot plots). Then CD127 and CD26 were used to identify five subsets within the CD4^−^ lymphocyte compartment (right dot plot). (**b**) Percentage of CD4^−^CD26^+^ or CD4^−^CD126^+^ cells from healthy controls (HC; N = 32), rhinitis (R; N = 44), allergic asthma (AA; N = 100) and non-allergic asthma (NAA; N = 92). (**c**) Percentage (median ± IQR1-3) of CD26^−^CD127^−^, CD26^int^CD127^+^, and CD26^high^CD127^+^ cells amongst CD4^−^ lymphocytes in HC, R, AA, and NAA. (**d**) Composition of CD4^−^ lymphocyte subsets based on CD3 (T), CD8 (Tc), CD19 (B), and CD56 (NK, NKT) antigens (Data from 3 representative donors). (**e**) Phenotypic analysis of CD4^−^ subsets based on CD45RA, CCR7, CD127, CD28, and CD27 markers (data from 3 representative donors). Statistically significant differences between groups are indicated (Kruskal-Wallis test: *P* < 0.05). In order to make the figure more understandable, sections (**c**,**d** and **e**) show only those CD4^−^ subsets gated in (**a**) (right dot plot) where differences between groups were observed (CD26^−^CD127^−^, CD26^int^CD127^+^, and CD26^high^CD127^+^ cells).

Next, we also decided to analyse the population of CD4^−^ lymphocytes (Fig. 3a; left and middle dot plots). This cell compartment is more heterogeneous and includes γδ-T, B, NK, and NKT lymphocytes. Analogously to what happened with Tlow cells, the percentage of CD4^−^ lymphocytes was negatively correlated with the percentage of CD26^+^/CD126^+^ cells in this subset (Supplementary Table S1). Moreover, along with the strong correlation between the expression levels of CD26 and CD126 on CD4^−^ lymphocytes (Fig. 1b), we also detected a decreased proportion of CD4^−^CD26^+^ and CD4^−^CD126^+^ cells in NAA compared to AA and R patients (Fig. 3b). Additionally, both CD4^−^CD26^+^ and CD4^−^CD126^+^ cells had a negative correlation with age (r_s_ = −0.482, *P* < 0.001; r_s_ = −0.508, *P* < 0.001, respectively).

To further characterize CD4^−^ lymphocytes, these cells were subdivided in five subpopulations according to CD26 and CD127 levels: CD26^int/high^CD127^−^, CD26^−^CD127^−^, CD26^−^CD127^+^, CD26^int^CD127^+^, and CD26^high^CD127^+^ cells (Fig. 3a). As Fig. 3c shows, we only detected an increment of double negative cells (CD26^−^CD127^−^) in NAA compared to AA, which was accompanied by a reduction of both CD26^int^CD127^+^ and CD26^high^CD127^+^ lymphocytes. These two last CD4^−^ subsets were rather homogeneous in terms of cell composition, exhibiting a high proportion (>80–90%) of cytotoxic T cells (Tc; CD3^+^CD8^+^) (Fig. 3d). In contrast, the composition of CD4^−^CD26^−^CD127^−^ lymphocytes was more complex and included Tc, B (CD19^+^) and CD56^+^ cells (NK, NKT, or γδ-T lymphocytes) (Fig. 3d).

Regarding the CD45RA and CCR7 markers, both CD26^+^ subpopulations (CD26^int^CD127^+^ and CD26^high^CD127^+^) displayed similar percentages of positive cells (Fig. 3e). Nevertheless, we noticed a decrease of CD45RA levels (mean fluorescence intensity/MFI) in CD26^high^ cells (41.4 ± 4.2) compared to the CD26^int^ subset (342.1 ± 198.2). This finding supports a differentiation of naïve cells (CD26^int^) towards a memory-like (CD26^high^) phenotype, compatible with the abundance of co-stimulatory molecules (CD27 and CD28) in both subsets (Fig. 3e). In contrast, only a small percentage of CD4^−^CD26^−^CD127^−^ cells were CD27^+^, CD28^+^, or CCR7^+^. Strikingly, they showed a CD45RA^high^ phenotype (Fig. 3e). Altogether, these characteristics define a T_EMRA_-like population within CD4^−^CD26^−^CD127^−^ lymphocytes. Similar results were obtained when CD4^−^ lymphocytes were segregated based on CD126 vs CD127 levels (Supplementary Fig. S3).

### The CD26^−/low^ subpopulation of γδ-T lymphocytes is augmented in non-allergic compared to allergic asthmatics

**Figure 4:**
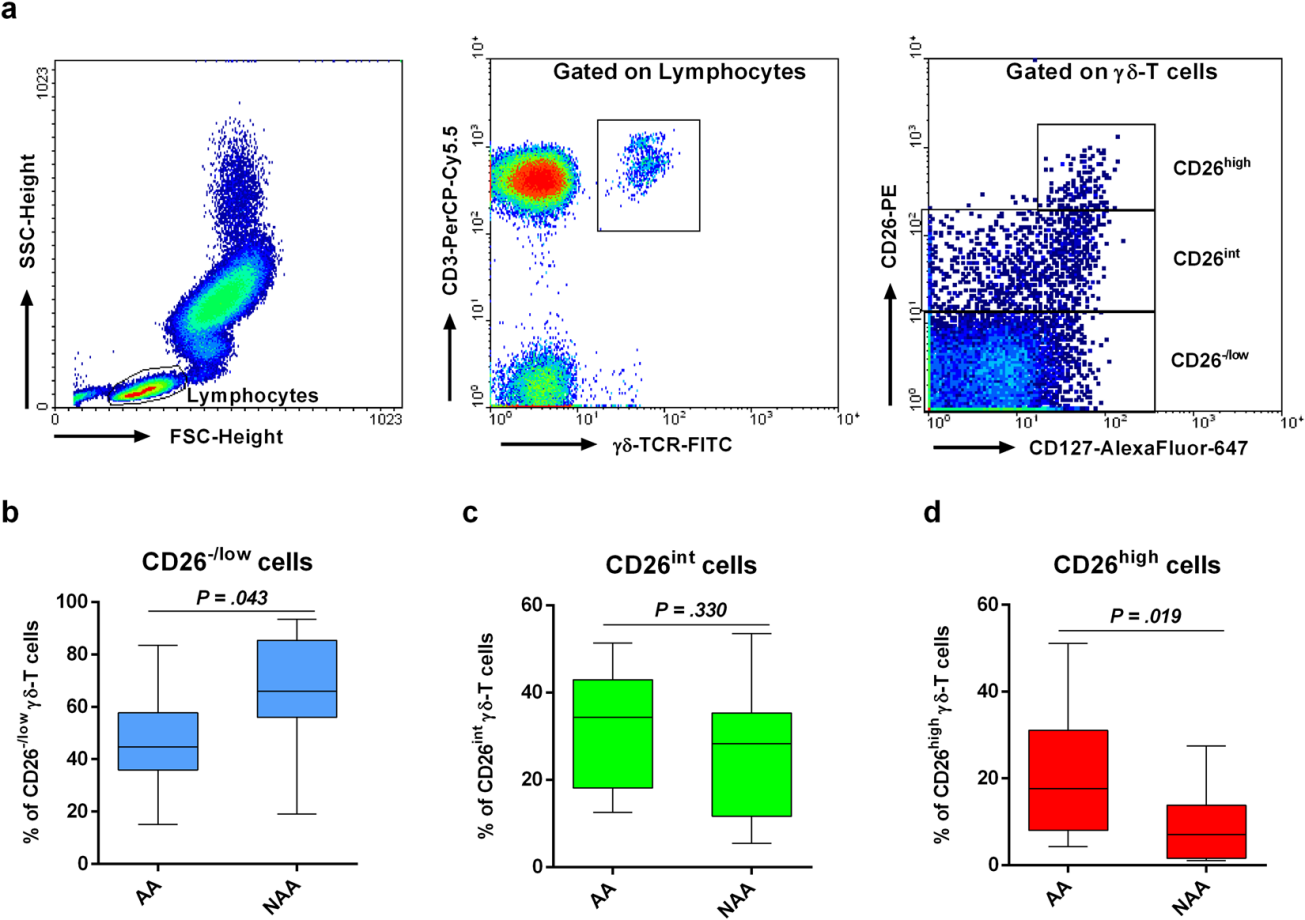
Non-allergic asthmatics exhibit augmented proportions of CD26^−/low^ γδ-T lymphocytes compared with allergic asthmatics. (**a**) Gating strategy for CD3^+^γδ-TCR^+^ lymphocytes (γδ-T cells). After gating, expression of CD127 and CD26 was used to identify three subsets (CD26^−/low^CD127^−/+^, CD26^int^CD127^+^, and CD26^high^CD127^+^) in γδ-T cells (right dot plot). (**b**–**d**) Percentage (median ± IQR1-3) of CD26^−/low^CD127^−/+^ (**b**), CD26^int^CD127^+^ (**c**), and CD26^high^CD127^+^ cells (**d**) amongst γδ-T lymphocytes in allergic (AA; N = 12) and non-allergic (NAA; N = 12) asthmatics. Statistically significant differences between these two groups of donors are indicated (t-test: *P* < 0.05).

To ascertain the specific lymphocyte subset within the CD4^−^ compartment showing altered proportions in NAA compared to AA and to limit possible cofounding effects (e.g. age, gender), we analysed blood samples from a second cohort study limited to AA and NAA donors (n = 12/each) with similar age and M/F proportions. The new analysis focused on CD8^+^ T (CD3^+^CD8^+^), NK (CD3^−^CD56^+^), NKT (CD3^+^CD56^+^), B (CD19^+^), and γδ-T cells (TCRγδ^+^CD3^+^) (Supplementary Fig. S4). There was no evidence for expansion of these circulating populations in NAA. However, this new study revealed an increased proportion of CD26^−/low^ γδ-T lymphocytes (and an opposite pattern for CD26^high^ γδ-T cells) in NAA compared to AA (Fig. 4). Once again, the percentage of γδ-T cells correlated inversely with the percentage of CD26^+^ cells within this subset (*r*_*s*_ = −0.460, *P* = 0.024). Interestingly, there was also a negative correlation between the percentages of γδ-T cells and B lymphocytes (*r*_*s*_ = −0.602, *P* = 0.002).

### CD26 could act as a negative regulator of T-cell proliferation

**Figure 5:**
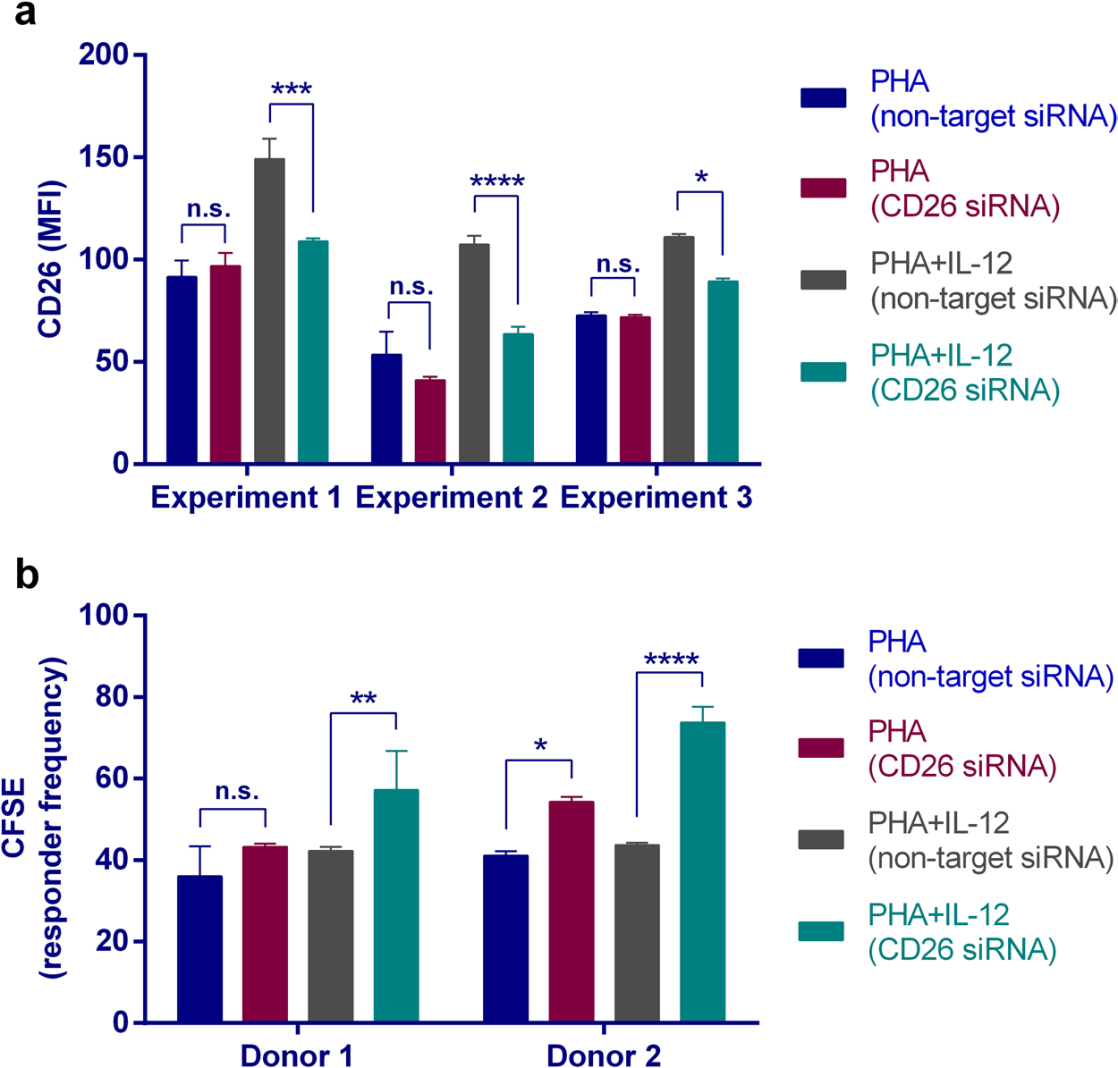
siRNA mediated depletion of CD26 mRNA leads to enhanced T-cell proliferation. PBMCs from healthy subjects were isolated and placed in culture for 6 days in 96 round-well plates. To promote T-cell division, Accell culture medium was supplemented with PHA ± IL-12. Besides, a CD26-specific or a non-targeting Accell siRNAs pool was also used. (**a**) Expression of CD26 (MFI; mean fluorescence intensity) on PBMCs was assessed by flow cytometry. Three representative assays are shown. 2-way ANOVA with Tukey’s multiple comparison test: *P < 0.05, ***P < 0.001, ****P < 0.0001; n.s., non-significant. (**b**) PBMCs from 2 representative donors were labelled with CFSE and T-cell proliferation induced by PHA ± IL-12 was assessed by CFSE-dilution assays. Responder frequency is the percentage of T lymphocytes that divided at least once. 2-way ANOVA with Tukey’s multiple comparison test: *P < 0.05, **P < 0.01, ****P < 0.0001; n.s., non-significant.

CD26 down-modulation on T lymphocytes from both AA (CD4^+^ T cells) and NAA (CD4^−^ γδ-T cells) patients could be: a) a consequence of different mechanisms that simply reduce the amount of this protein on the cell surface, like, for example, the dilution of CD26 molecules (half-life > 48 h) throughout the successive cytokinesis rounds; or b) a necessary condition to initiate the proliferation/differentiation programme of naïve or memory T cells. To distinguish between both possibilities, we tested the effect of RNA interference (RNAi) on the *DPP4* gene during the proliferative response of T lymphocytes to mitogenic triggers. Peripheral blood mononuclear cells (PBMCs) were CFSE-labelled and cultured *in vitro* with either non-target or CD26/DPP4-specific Accell siRNAs. As CD26 up-regulation during T cell activation was mainly derived from the translocation of this protein from intracellular stores toward the cell surface, T-cell division was stimulated with phytohemagglutinin P (PHA) in the presence or absence of IL-12, a cytokine that promotes CD26 mRNA translation. Furthermore, it was required to extend the *in vitro* culture incubation for 6 days to observe the inhibitory effect of the CD26-specific siRNA on protein levels. As expected, CD26-specific siRNAs down-modulated the expression of CD26, but only in IL-12-stimulated PBMCs (Fig. 5a). After verification of compliance with CD26 down-modulation by RNAi, we estimated the percentage of cells that divided at least once. As Fig. 5b shows, those T cells where *DPP4* gene silencing was more intense (i.e. IL-12-costimulated) were the ones showing an increase in the proliferation rate.

### CD126/IL-6Rα down-modulation in neutrophils, monocytes, and lymphocytes is associated to disease severity and asthma control

**Figure 6:**
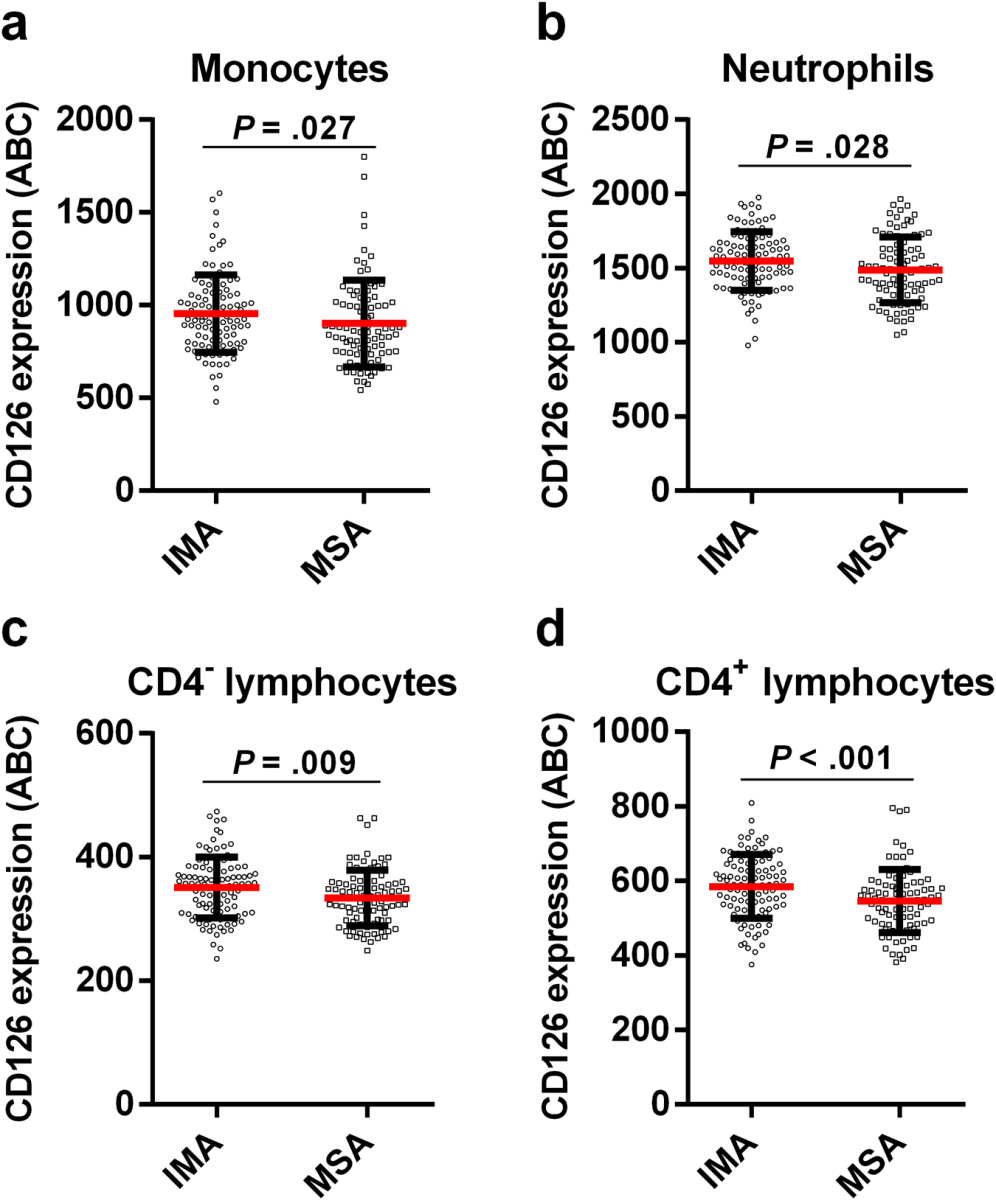
Generalised down-modulation of CD126 in leukocytes from patients with moderate-severe asthma. Monocytes (**a**), neutrophils (**b**), and lymphocytes (**c**,**d**) were gated on a flow cytometry FSC/SSC plot. Afterwards, lymphocytes were further subdivided into CD4^−^ (**c**) and CD4^+^ (**d**) cells. The number of CD126 antibodies bound per cell (ABC; median ± IQR1-3; see Material and Methods section) was measured on every population (**a**–**d**) in intermittent-mild (IMA; N = 102) and moderate-severe (MSA; N = 90) asthma patients. Statistically significant differences between these two groups of donors are indicated (t-test: *P* < 0.05).

Asthma severity could be responsible for changes in pulmonary and inflammatory parameters. To assess this, we segregated asthma patients into moderate-severe (n = 90) and intermittent-mild (n = 102) asthmatics; asthma control degree was also considered (Table 1). As expected, FEV1 and FEV1/FVC were decreased in moderate-severe and uncontrolled asthmatics (Supplementary Table S2), regardless of phenotype. Eosinophils count was also augmented in moderate-severe and badly-controlled asthmatics (Supplementary Table S2), although the statistical significance is only maintained in AA after segregation according to the phenotype. In contrast, badly-controlled NAA was only characterized by an increased neutrophils count (*P* = 0.032; data not shown). Interestingly, there was a decrease in the expression of CD126 in many leukocyte subsets (monocytes, neutrophils, CD4^−^ and CD4^+^ lymphocytes) as the severity was higher (Fig. 6). This finding extends to Teff, Treg, and Tlow lymphocytes. Similar decreased levels were obtained for CD126 on leukocytes from badly-controlled asthmatics. Furthermore, we did not observe changes in CD26 levels on most leukocyte subpopulations with asthma severity, although the percentage of CD26^+^ Tregs was higher in moderate-severe NAA patients (Supplementary Fig. S5). Finally, the percentage of CD4^+^ lymphocytes was higher in badly-controlled AA (*P* = 0.006; data not shown).

## Discussion

This study shows an intense correlation between the expressions of CD26/DPP4 and CD126/IL-6Rα, two molecules that identify naïve (CD26^int^ and/or CD126^+^ cells), memory (CD26^high^ and/or CD126^+^), and highly differentiated effector subsets (T_EM_ and T_EMRA_ cells; CD26^−/low^ and/or CD126^−^). Precisely, the last subset is expanded in both groups of asthma patients: AA (CD4^+^ Tlow cells) and NAA (γδ-T cells). Probably as a result of this expansion, there is a reduction of sCD26 concentration in plasma that is shared by AA and NAA patients. Moreover, CD26 could be relevant to slow the rate of cell division of naïve or central-memory T lymphocytes and, conversely, its down-modulation necessary for rapid proliferation and differentiation into effector cells. Finally, down-regulation of CD126 on leukocytes may be related to asthma severity.

CD26 is an activation marker^18^ that identifies TH_1_ and especially TH_17_ cells^11^. Our results show an increase of “activated” CD4^+^ cells (CD26^+^) in asthma, in agreement with published work^19^. Despite the IL-13-dependent up-regulation of CD26/DPP4 on human bronchial epithelial cells^43^ or the potential role of TH_17_ cells in NAA^8,44^, both AA and NAA patients express similar levels of CD26 on CD4^+^ T cells. A possible explanation for this observation is that following antigen presentation to naïve CD4^+^ T cells in lymph nodes, these lymphocytes proliferate, differentiate, and move back to the peripheral circulation, where they still have an early differentiation state (CD26^high^; TH_17_ ≫ TH_1_ > TH_2_) and an extended half-life compared to innate leukocytes (CD26^−/low^; eosinophils, neutrophils) (Supplementary Table S3). Therefore, these circulating TH cells will still require the down-modulation of CD26 to become T_EM_/T_EMRA_ cells and migrate into sites of inflammation; a similar event would occur with IL-6Rα/CD126 on T lymphocytes to favour the proinflammatory *trans*-signalling pathway.

Three major TH subsets coexist in circulation: Treg (CD25^high^CD26^low^CD127^low^), “conventional” Teff (CD25^low^CD26^high^CD127^high^), and Tlow cells (CD25^−/low^CD26^−/low^CD127^−/low^). The latter is a highly differentiated counterpart of “conventional” Teff cells. CD4^+^ Tlow cells are expanded in AA^20^ and their abundance is negatively correlated with CD26/CD126 levels, as it happens for other subpopulations (e.g. CD26^−/low^ γδ-T lymphocytes). Despite the small expansion of the Tlow cell compartment, this might be relevant for AA pathogenesis considering two facts: (a) total lymphocytes, instead of antigen-specific cells, have been measured; and (b) patients were in stable phase (i.e. absence of exacerbations for 4 weeks before sample collection). On the other hand, even though there is a negative association between the percentage of CD26^+^/CD126^+^ cells and the percentage of Tlow cells, this does not necessarily imply CD26-dependent causation. Therefore, we performed T cell proliferation assays after siRNA-mediated depletion of CD26 mRNA. These results show a negative regulatory role for CD26 in T cell proliferation^15^. Thus, our data agree with the study of Yan *et al*. working with ovalbumin-induced CD26^−/−^ C56BL/6 animals^45^, or Stephan *et al*. showing that oral administration of DPP4-inhibitors aggravates the airway inflammation in a rat model of asthma^46^.

A reduced expression of CD27, CD28, and CCR7 is the hallmark of highly-differentiated effector cells (T_EM_ or T_EMRA_). Tlow cells fulfil these criteria; therefore, these lymphocytes might be part of a pool of “conventional” CD4^+^ Teff cells that remains upon antigen clearance and develop into senescent lymphocytes with short telomeres, low proliferative capacity, and presence of cytotoxic molecules^47^. Thus, in line with other works^17,28^, expression of CD26/DPP4 and CD126/IL-6Rα would identify cells with a naïve (CD45RA^+^, CD62L^+^, CCR7^+^) or a central-memory (CD45RO^+^, CD62L^+^, CCR7^+^) phenotype, while reduced levels would distinguish CD4^+^ T subsets expanded in AA with an T_EM_ or T_EMRA_ phenotype. Similarly, we found augmented proportions of peripheral blood CD4^−^ γδ-T cells with a CD26^−^CD126^−^CD127^−^ phenotype (likely V𝛿2/Vγ9^+^)^48^ in NAA compared to AA. Several studies have described the presence of different circulating V𝛿2/Vγ9^+^ T cells (naïve/CD45RA^+^CD27^+^, T_CM_/CD45RA^−^CD27^+^, T_EM_/CD45RA^−^CD27^−^, and T_EMRA_/CD45RA^+^CD27^−^)^49,50^, which is compatible with the existence of CD26^−^ (T_EM_/T_EMRA_), CD26^int^ (naïve), and CD26^high^ (T_CM_) γδ-T subsets. Thus, an enlarged population of CD26^−^ γδ-T lymphocytes (i.e. T_EM_/T_EMRA_) with a preferential production of TH_17_ or TH_1_ cytokines^51^ in NAA patients could explain the enhanced airway inflammation and the inverse relationship between γδ-T cell and B-cell proportions. However, it has been previously reported that most of circulating γδ-T cells are CD126^−^gp130^−^ (i.e. they are not IL-6-responders)^52^, which raises the question about how they can become IL-17-producers.

Another caveat is if those phenotypic changes on T lymphocytes could be mirrored in serum samples. Different circulating molecules were measured (TGF, TNF, sCD25, sCD26) (Table 1; Fig. 1), but most of them remained unaltered. We did not quantify “soluble” IL-6R/CD126 (sIL-6R/sCD126), but several authors reported higher levels in stable asthma and especially upon flare-ups due to mechanisms involving sheddases (e.g. ADAM10/17)^53–55^, spliceosomes^53,56^, or vesicles^54^. In contrast, changes in sCD26 levels in asthma remain almost unexplored. Lun *et al*. reported an elevation of sCD26 in AA patients linked to the activated phenotype (CD26^high^) of CD4^+^ T lymphocytes^19^. However, reduced sCD26 levels have been recently described in severe asthmatics^57^ or a low eosinophilic TH_2_^low^ severe asthma endotype^58^. Our results are in agreement with these last works and support a rather generalized (AA and NAA) sCD26 down-modulation. The underlying mechanism is likely the expansion of CD26^−^ T subsets^20^ with a T_EM_ or T_EMRA_ phenotype: CD4^+^ T cells in AA and CD4^−^ γδ-T cells in NAA. Reduced levels of CD26 on lymphocytes and the extracellular compartment could be concomitant with the loss of caveolin-1 (a CD26 ligand) in bronchial epithelial cells and monocytes from asthmatics^15^. Moreover, the decrease of CD26 levels may be important for the bioavailability of soluble factors (e.g. chemokines, adenosine) and to promote cell functions like proliferation, chemotaxis, and migration toward inflamed tissues^15^.

Treg cell function has been described as impaired in asthma^59^. Although we did not find deregulation of Treg numbers, they showed increased CD26 expression in asthmatic patients. This is relevant because CD39 is an ecto-enzyme expressed by CD26^−^ Treg lymphocytes^12,60^ and involved in adenosine (Ado) production^61^. Ado is an immune-regulatory molecule whose synthesis is counteracted by adenosine deaminase (ADA), an ecto-enzyme anchored to CD26^62,63^. Therefore, a CD26^high^ phenotype in Tregs could decrease local Ado concentration and exacerbate disease severity^64^. Indeed, the percentage of CD26^+^ Treg cells in NAA was higher in moderate-severe patients than intermittent-mild subjects. The percentage of CD127^+^ Treg cells, a phenotype correlated with a diminished suppressive capacity^65^, was also augmented in asthmatics compared to rhinitis and HC. However, future studies including the assessment of Treg function in NAA and AA will be necessary.

Asthma severity is also influencing CD126 levels on CD4^+^ T cells, neutrophils and monocytes. IL-6 acts via either IL-6R (classic-signalling) or sIL-6R/sCD126 (*trans*-signalling)^22^. Contrary to the anti-inflammatory role of the first pathway^28^, the *trans*-signalling route allows CD126^−^CD130^+^ cells to respond to IL-6^27^ and is important in asthma through the maintenance of TH_17_ cells or the inhibition of T-cell apoptosis^66^. Naïve CD4^+^ T cells down-modulate IL-6R upon TCR-mediated activation, probably due to protein shedding^28^. This release mechanism has been observed in asthma, and sIL-6R levels have been directly associated with IgE levels, but negatively with lung function^67^. Therefore, reduction of CD126 expression in monocytes, neutrophils, and CD4^+^ cells from moderate-severe patients highlights the role of IL-6 *trans*-signalling in asthma severity.

In conclusion, our data provide evidence that both asthma phenotypes share common immune-pathologic mechanisms, with expansion of CD26^−/low^ subsets in AA (CD4^+^ Tlow or “highly-differentiated” Teff cells) and NAA (CD4^−^ T cells; γδ-T lymphocytes) and down-modulation of additional surface molecules (IL-6Rα/CD126, CD27, CD28, IL-7Rα/CD127, CCR7) to produce differentiated effector subsets and extracellular sCD26 reduction. This CD26/sCD26 down-modulation and the potential role in T-cell proliferation should be considered in the light of clinical usage of DPP4 inhibitors and anti-CD26 antibodies.

## Material and Methods

### Subjects

Adult patients with asthma or allergic rhinitis were recruited from hospital consultations for Pneumology in Galicia (Spain) between 2014 and 2016. The diagnosis of different asthma phenotypes and allergy was confirmed according to Global Strategy for Asthma Management and Prevention (GINA 2006, http://www.seicap.es/documentos/archivos/GINA2006general.pdf) criteria for at least one year prior to study initiation. A positive skin prick test and the presence of allergen-specific IgE were used to confirm sensitization in allergic patients. Lung function parameters (forced expiratory volume in the 1st second (FEV1), forced vital capacity (FVC), and FEV1/FVC ratio) as well as eritro-sedimentation rate (ESR) were also analysed. All asthmatics were in a stable phase of the disease (i.e. absence of exacerbations for at least 4 weeks before sample collection). Healthy donors were subjects without allergy or systemic diseases, who were scheduled for minor surgeries (orthopedic surgery or inguinal hernia). A second cohort of patients was also included with 12 patients with AA (M/F proportion: 6/6; age: 52.75 ± 14.12) and 12 patients with NAA (M/F proportion: 6/6; age: 61.00 ± 10.71), recruited from hospital consultations for Pneumology in Galicia, Spain. The research project was approved by the Ethics Committee of Clinical Research of Galicia (2011/001), Spain, all subjects signed an informed consent, and all research was performed in accordance with the relevant guidelines and regulations.

### Biochemical determinations

Biochemical determinations and nucleated cell counting were performed by using an ADVIA^®^1650 analyser (SIEMENS Healthcare Diagnostics S.L., Berlin, Germany) and an ADVIA^®^2120 haematology counter (SIEMENS Healthcare Diagnostics S.L., Berlin, Germany), repectively. Serum TGF-β1 levels were measured using ELISA plates (Human TGF-beta1 Platinum ELISA; ref. BMS249/4TEN; eBioscience) following commercial guidelines. Optical densities were assayed at 450 nm (Labsystem Multiscan MS), and protein concentration was calculated from standard curves.

### Flow cytometry assays

Venous peripheral blood from each donor was collected in EDTA tubes (BD Vacutainer K2E). Then, FITC, PE, PE-Cy7, PerCP-Cy5.5 or AlexaFlour-647-labelled mouse IgG1 κ isotype antibodies (BD Bioscience) or specific antibodies were incubated with cells for 30 min in FACS buffer (PBS, pH 7.4, 2% FBS). We used BD FACS^TM^ Lysing Solution (15 min; room temp.) to lyse red blood cells before sample collection. Finally, 200000 events were acquired on a BD FACSort^TM^ flow cytometer and data were analysed using WinMDI 2.9 software (Joseph Trotter, La Jolla, CA. USA). A list of antibodies is shown in Supplementary Table S4. Isotype antibodies were used to determine the non-specific binding of the antibodies and therefore to set a threshold value to identify negative and positive populations. Single stained lymphocytes were used for fluorescence compensation.

Flow cytometry data are presented as either “percentage of positive cells” or “number of antibodies per cell” (ABC) instead of mean fluorencence intensity (MFI). We used the BD Quantibrite™ Beads PE kit (Fluorescence Quantitation Kit; BD Bioscience) to estimate ABC according to manufacturer instructions. In brief, we ran a BD Quantibrite PE tube with the same instrument settings as the assay. Therefore, MFI values in FL2 were converted into the number of PE molecules bound per cell. Finally, this number of PE molecules/cell was changed into ABC values by using known ratios of PE to antibodies. We took advantage of this transformation to minimise as much as possible the inter-day variation related to the working conditions of the flow cytometer.

### *In vitro* proliferation assays and CD26 mRNA silencing

PBMCs were placed in RPMI 1640 at a cell density of 10^7^ cell/mL and incubated with 5 μM CFSE for 8 min at RT in the dark. Then, FBS was added to stop the reaction and cells were thoroughly washed with RPMI 1640 before cell counting. Cell cultures were set up at 0.25 × 10^6^ cells/mL in 96-well microplates (round-wells). Accell delivery media (ref. B-005000-500; Dharmacon) was used to culture these cells under non-serum conditions. The Accell delivery medium was supplemented or not with 1 μg/ml PHA (±2 ng/ml IL-12), in the presence of either DPP4-specific or non-targeting Accell siRNAs pools (Dharmacon). To achieve a partial gene silencing we used a commercial Accell SMART pool of 4 short interfering RNA (siRNA) designed to target the mRNA encoded by the human *DPP4* gene (ref. E-004181-00-0005; Dharmacon); these siRNAs were designed to minimize the off-target effects. Besides, we also used two non-target siRNAs: a) an Accell green non-targeting siRNA (ref. D-001950-01-05; Dharmacon), which is a fluorescent unspecific siRNA used for assessment of Accell siRNA passive delivery effectiveness; b) a negative control Accell non-targeting siRNA pool of four siRNAs (ref. D-001910-10-05; Dharmacon) to control the background response to siRNA. All these siRNAs were initially resuspended at 100 μM by using a 1X siRNA buffer (ref. B-002000-UB-100; Dharmacon). Strikingly, the working concentration suggested by the manufacturer (1 μM) induced high cell mortality. Therefore, we titrated down the concentration of siRNA by using the Accell green non-targeting siRNA. A final concentration of 0.02 μM was selected to carry out the final experiments. This concentration was enough to label > 98% of cells and allow a high cell viability (>95%).

Upon 6 days of *in vitro* culture, CFSE fluorescence and the number of cell divisions were measured by flow cytometry (Supplementary Fig. S6), as well as the amount of CD26 protein with both specific anti-CD26 and isotype antibodies. Each condition was tested several times (n = 3–5 technical replicates). Unlabelled cells served as negative controls in cell proliferation assays. The calculated responder frequency (Rf) is the percentage of responder T cells that divided at least once (Supplementary Fig. S6).

### Statistics

Descriptive data are presented as median (interquartile range; IQR1-3). To assess the changes between asthmatic groups, rhinitis, and healthy donors for non-normally distributed variables we used the Kruskal–Wallis test followed by Dunn’s multiple comparison test. Spearman’s test was used to measure association between these variables. For CFSE proliferation studies, a two-way ANOVA followed by a Tukey’s multiple comparison test were used. Finally, *t-test* was performed with data from Fig. 2c to assess changes in normally distributed variables between moderate-severe allergic asthmatics and healthy subjects. Mann-Whitney U test was used to assess changes in non-normally distributed variables. All analyses were conducted using GraphPad Prism 6.0 (GraphPad Software, Inc., San Jose, California, USA). The statistical significance was defined as *P* < 0.05.

## Supplementary information

Supplementary Information

## Data Availability

The datasets analyzed are available from the corresponding author on justifiable request, and not publicly available due to protection of participant confidentiality.
