# Supplementary material for: Expansion of different subpopulations of CD26−/low T cells in allergic and non-allergic asthmatics
Source: Sci Rep. 2019 May 17;9:7556. doi: 10.1038/s41598-019-43622-8 (PMC6525268; doi:10.1038/s41598-019-43622-8)
Supplement: Supplementary file 1 — Supplementary Information [file 41598_2019_43622_MOESM1_ESM.pdf]

Expansion of different subpopulations of CD26$^{-/low}$ T cells in allergic and non-allergic asthmatics.

Authors

Juan José Nieto-Fontarigo[1], Francisco Javier Salgado*[1], María Esther San-José[2], María Jesús Cruz[3], Luis Valdés[4], Amparo Pérez-Díaz[5], Pilar Arias[1], Montserrat Nogueira[1], Francisco Javier González-Barcala[6].

Affiliations:

[1]Department of Biochemistry and Molecular Biology, Faculty of Biology-Biological Research Centre (CIBUS), Universidade de Santiago de Compostela, Santiago de Compostela, Spain.

[2]Clinical Analysis Service, USC University Hospital Complex (CHUS), Santiago de Compostela, Spain.

[3]Department of Respiratory Medicine-Hospital Vall d'Hebron, Universitat Autònoma de Barcelona, Barcelona, Spain. Spanish Biomedical Research Networking Centre-CIBERES.

[4]Department of Medicine-University of Santiago de Compostela; Department of Respiratory Medicine-University Hospital of Santiago de Compostela; Health Research Institute of Santiago de Compostela (IDIS).

[5]Drug Screening Platform/Biofarma Research Group, Molecular Medicine and Chronic Diseases Research Center, University of Santiago de Compostela, Santiago de Compostela, Spain.

[6]Department of Medicine-University of Santiago de Compostela, Department of Respiratory Medicine-University Hospital of Santiago de Compostela, Spanish Biomedical Research Networking Centre-CIBERES; Health Research Institute of Santiago de Compostela (IDIS).

Corresponding author:

Francisco Javier Salgado Castro. Biology of Lymphocyte Group (BioLympho). Department of Biochemistry and Molecular Biology, Faculty of Biology-Biological Research Centre (CIBUS),

Universidade de Santiago de Compostela, Campus Vida, C/ Lope Gómez de marzoa s/n, Santiago de Compostela, Spain. E-mail: Phone: +34 881816941.

Supplementary Tables: 4

Supplementary Figures: 6

Supplementary Table S1. Correlation of CD26 and CD126 expression with the % of different leukocyte subsets.

	Correlation between the % of cells and the % of CD26+ cells in each subpopulation		Correlation between the % of cells and the % of CD126+ cells in each subpopulation	
	r_s	*P*	r_s	*P*
**Eosinophils**	**-.181**	**.003**	**.205**	**< .001**
**Monocytes**	**-.206**	**< .001**	-.085	.168
**Neutrophils**	.041	.500	.035	.564
**Lymphocytes**	-.103	.094	-.057	.354
**$CD4^-$ cells**	**-.312**	**< .001**	**-.353**	**< .001**
**$CD4^+$ cells**	**.367**	**< .001**	**.211**	**< .001**
**Teff cells**	**.469**	**< .001**	**.247**	**< .001**
**Treg cells**	.023	.702	-.070	.254
**Tlow cells**	**-.342**	**< .001**	**-.360**	**< .001**

Supplementary Table S2. Severity and control degree of the study patients.

	IMA	MSA		CA	UA
**N**	102	90		158	34
**Age (mean (range))**	44 (20-72)	41 (18-68)		45 (18-72)	45 (20-68)
**Sex (M/F)**	33/69	35/55		59/99	9/25
**FEV1 (%)**	105.0 (95.0-113.3)***	86.5 (67.0-100.0)		99.0 (87.0-111.0)*	82.0 (60.7-100.0)
**FEV1/FVC (%)**	77.5 (72.5-81.2)***	70.3 (59.5-78.5)		76.4 (69.0-80.5)*	70.0 (56.4-76.8)
**Neutrophils (10^3 cells/µL)**	3.60 (3.09-4.23)	3.62 (2.88-4.40)		3.58 (3.07-4.23)	4.17 (2.78-5.00)
**Lymphocytes (10^3cells/µL)**	1.97 (1.65-2.25)	1.97 (1.64-2.54)		1.95 (1.60-2.26)	2.02 (1.86-2.74)
**Monocytes (10^3 cells/µL)**	0.39 (0.30-0.48)	0.39 (0.32-0.49)		0.38 (0.30-0.49)	0.41 (0.32-0.48)
**Eosinophils (10^3 cells/µL)**	0.26 (0.17-0.39)*	0.33 (0.21-0.48)		0.27 (0.18-0.40)*	0.38 (0.21-0.63)
**Basophils (10^3 cells/µL)**	0.04 (0.02-0.05)	0.04 (0.03-0.05)		0.04 (0.02-0.05)	0.04 (0.03-0.06)
**IgE (IU/mL)**	45.5 (15.5-205.0)	84.0 (28.2-209.5)		79.0 (20.5-200.0)	62.0 (24.7-274.0)

CA, controlled asthmatics; IMA, intermittent-mild asthmatics; MSA, moderate-severe asthmatics; UA, uncontrolled asthmatics.
Data are presented as median value (IQR1-3), unless otherwise expressed.
Mann-Whitney U test was used to compare IMA vs. MSA and CA vs. UA. * $P < 0.05$; *** $P < 0.001$

Supplementary Table S3. Average expression of CD26 and CD126 molecules in different leukocyte subsets and the half-life of these cells in peripheral circulation.

	**Eosinophils**	**Monocytes**	**Neutrophils**	**Lymphocytes**
**Absolute count (x10^3 cells/mL)**	0.04-0.4	0.2-0.8	2.5-7.5	1.5-3.5
**% of $CD126^+$ cells***	63.4 (44.5-77.5)	78.5 (52.2-91.0)	99.5 (98.8-99.8)	55.3 (48.6-61.3)
**% of $CD26^+$ cells***	2.1 (1.4-3.2)	7.4 (3.5-17.6)	11.9 (5.0-27.7)	59.2 (54.4-66.4)
**Half-life in peripheral blood[#]**	8-18 h	22-24 h	6 h - 5 days	days-years

*Median value (IQR1-3).

[#]Data come from Pillay J, et al. Blood. 2010;116:625-7; Park YM, et al. Allergy Asthma Immunol Res. 2010;2:87-101; Patel AA, et al. J Exp Med. 2017;214:1913-1923; Robertson JM, et al. Immunol Rev. 2006;211:49-57.

Supplementary Table S4. List of monoclonal antibodies used in flow cytometry assays.

Target	Clone	Fluorophore	Provider	Cat. no
**CD3**	UCHT1	FITC	BD Pharmigen™	555332
**CD4**	RPA-T4	FITC	BD Pharmigen™	555346
**CD25**	M-A251	PE-Cy7	BD Pharmigen™	5135905
**CD127**	HIL-7R-M21	AlexaFluor-647	BD Pharmigen™	558598
**CD3**	UCHT1	PerCP-Cy5.5	BD Pharmigen™	560835
**CD8**	RPA-T8	PerCP-Cy5.5	BD Pharmigen™	560662
**CD19**	HIB19	PerCP-Cy5.5	BD Pharmigen™	561295
**CD27**	M-T271	PerCP-Cy5.5	BD Pharmigen™	560612
**CD28**	CD28.2	PerCP-Cy5.5	BD Pharmigen™	560685
**CD45RA**	HI100	PerCP-Cy5.5	BD Pharmigen™	563429
**CD56**	B159	PerCP-Cy5.5	BD Pharmigen™	560842
**CD197 (CCR7)**	150503	PerCP-Cy5.5	BD Pharmigen™	561144
**TCR-γ/δ**	B1	FITC	Biolegend	331207
**CD26**	TP1/19	PE	Immunostep	PE26 100T
**CD126 (IL-6Rα)**	REA291	PE	MACS Miltenyi Biotec	130-104-101

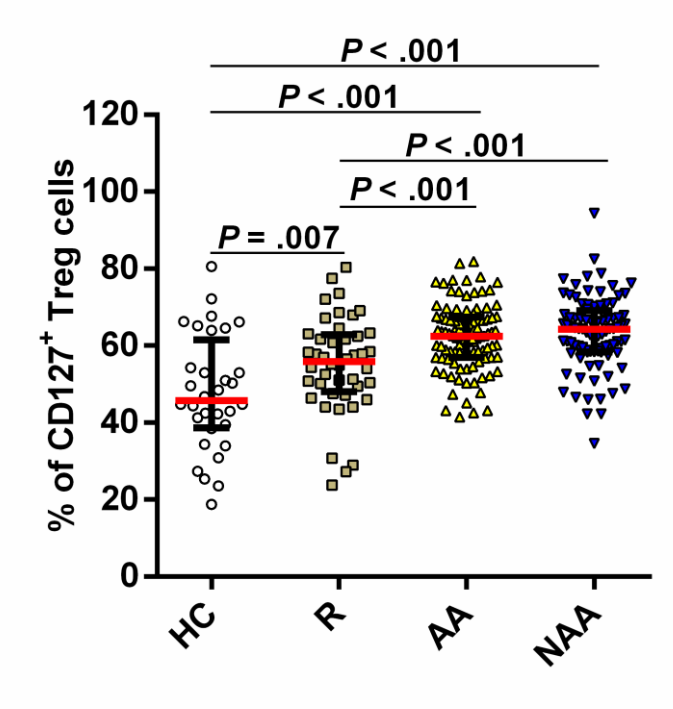

Supplementary Figure S1. Rhinitic and asthmatic patients show augmented levels of CD127$^+$ regulatory T cells. Lymphocytes from healthy controls (HC; N = 32) and patients with rhinitis (R; N = 44), allergic asthma (AA; N = 100), and non-allergic asthma (NAA; N = 92) were gated on FSC/SSC plots and further subdivided into CD4$^-$ and CD4$^+$ cells. Then, regulatory T cells (Treg) were identified amongst CD4$^+$ T lymphocytes according to CD25 and CD127 markers. Afterwards, the percentage of CD127$^+$ cells within the Treg compartment was measured. Statistically significant difference between the different groups of donors is indicated (*P* < .05).

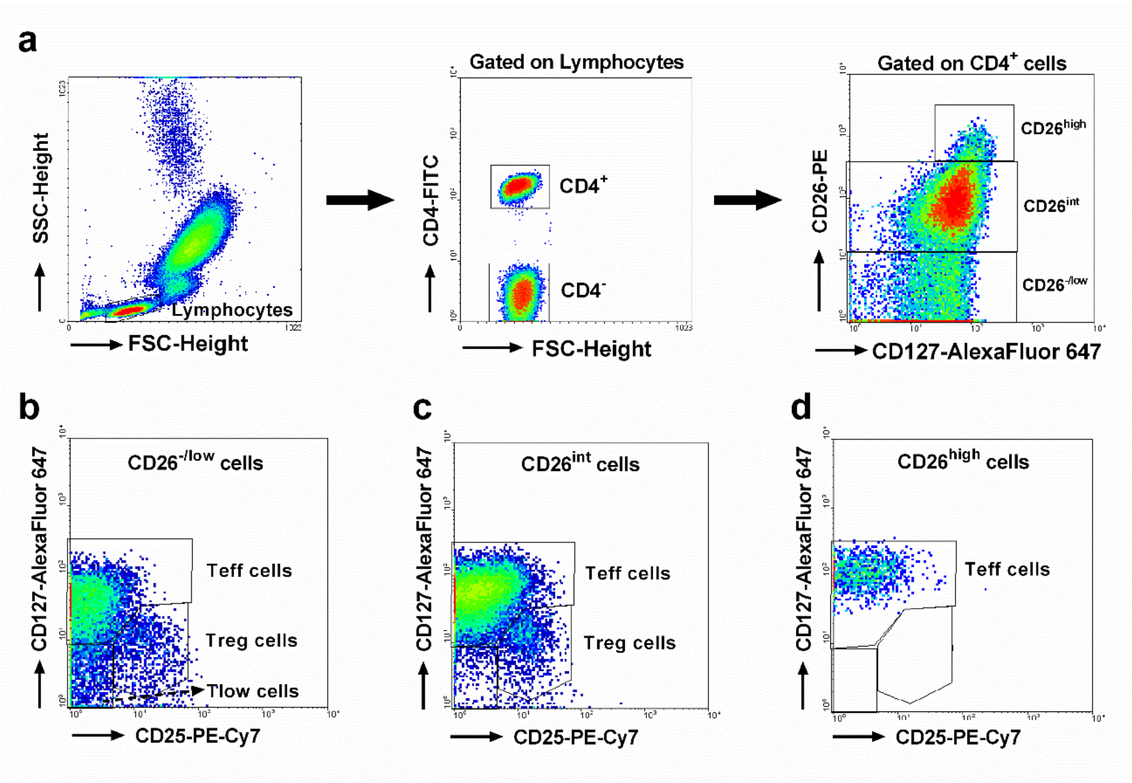

Supplementary Figure S2. Treg cells are distributed within CD26$^{-/low}$ and CD26^{int} subsets of CD4^{+} T cells. a) Lymphocytes from healthy controls (HC) and patients with rhinitis (R), allergic asthma (AA), and non-allergic asthma (NAA) were gated on FSC/SSC plots and further subdivided into CD4^{-} and CD4^{+} cells. Then, 3 subsets of CD4^{+} lymphocytes were identified based on CD26 and CD127 markers: CD26$^{-/low}$, CD26^{int} and CD26^{high}. As figures **b-d** point out, Treg cells (CD25^{high}CD127^{low}) are present in CD26$^{-/low}$ **(b)** and CD26^{int} **(c)** subsets of CD4^{+} T lymphocytes, but not amongst the CD26^{high} compartment of T helper cells **(d)**.

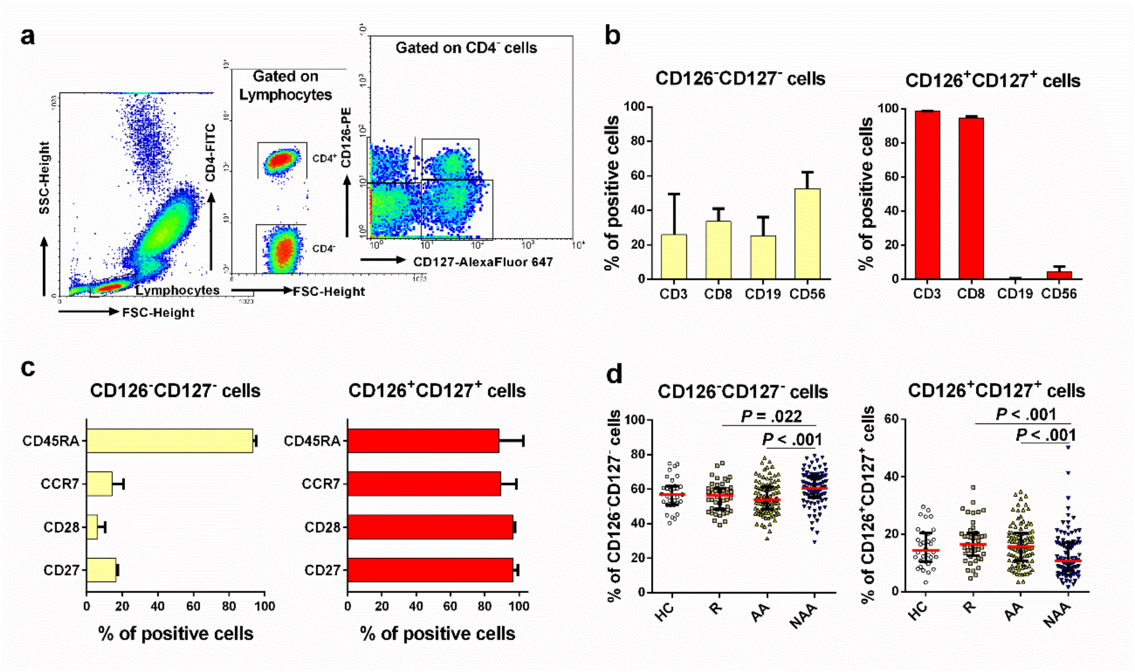

Supplementary Figure S3. CD4$^-$ lymphocytes with a CD126$^-$CD127$^-$ phenotype are increased in non-allergic asthmatics (NAA) compared to allergic asthmatics (AA). a) CD4$^-$ lymphocytes were identified through the gating strategy depicted (left and middle dot-plots), and expression of CD127 and CD126 was used to identify four subsets (right dot plot). **b)** Lymphocyte subset composition of CD4$^-$CD126$^-$CD127$^-$ and CD4$^-$CD126$^+$CD127$^+$ cells based on CD3 (T), CD8 (Tc), CD19 (B), and CD56 (NK, NKT) antigens. **c)** Phenotype of CD4$^-$ CD126$^-$CD127$^-$ and CD4$^-$CD126$^+$CD127$^+$ cells based on CD45RA, CCR7, CD127, CD28, and CD27 markers. We evidenced that the former CD26^{int}CD127$^+$ and CD26^{high}CD127$^+$ subsets were combined in one single CD4$^-$ population (CD126$^+$CD127$^+$ cells), with a homogeneous naïve-memory CD3$^+$ and CD8$^+$ phenotype. In contrast, the CD126$^-$CD127$^-$ subset comprised T$_{EMRA}$-like cells (CD45RA^{high}CCR7^{low}CD27^{low}CD28^{low}), but a less-defined CD4$^-$ lymphoid origin (B, NK, NKT, or γδ-T cells). In **b)** and **c)**, data were obtained from 3 representative donors. **d)** Percentage (median ± IQR) of CD126$^-$CD127$^-$ and CD126$^+$CD127$^+$ cells in CD4$^-$ lymphocytes from healthy controls (HC; N = 32) and patients with rhinitis (R; N = 44), allergic asthma (AA; N = 100), and non-allergic asthma (NAA; N = 92). Like CD4$^-$CD26$^+$CD127$^+$ lymphocytes, CD4$^-$CD126$^+$CD127$^+$ cells were reduced in NAA compared to AA, which was associated to the expansion of the double negative (CD126$^-$CD127$^-$) compartment in NAA. Only results from those subpopulations with significant differences between donor groups were shown. Statistically significant differences between groups of donors were indicated (Kruskal-Wallis test: $P < .05$).

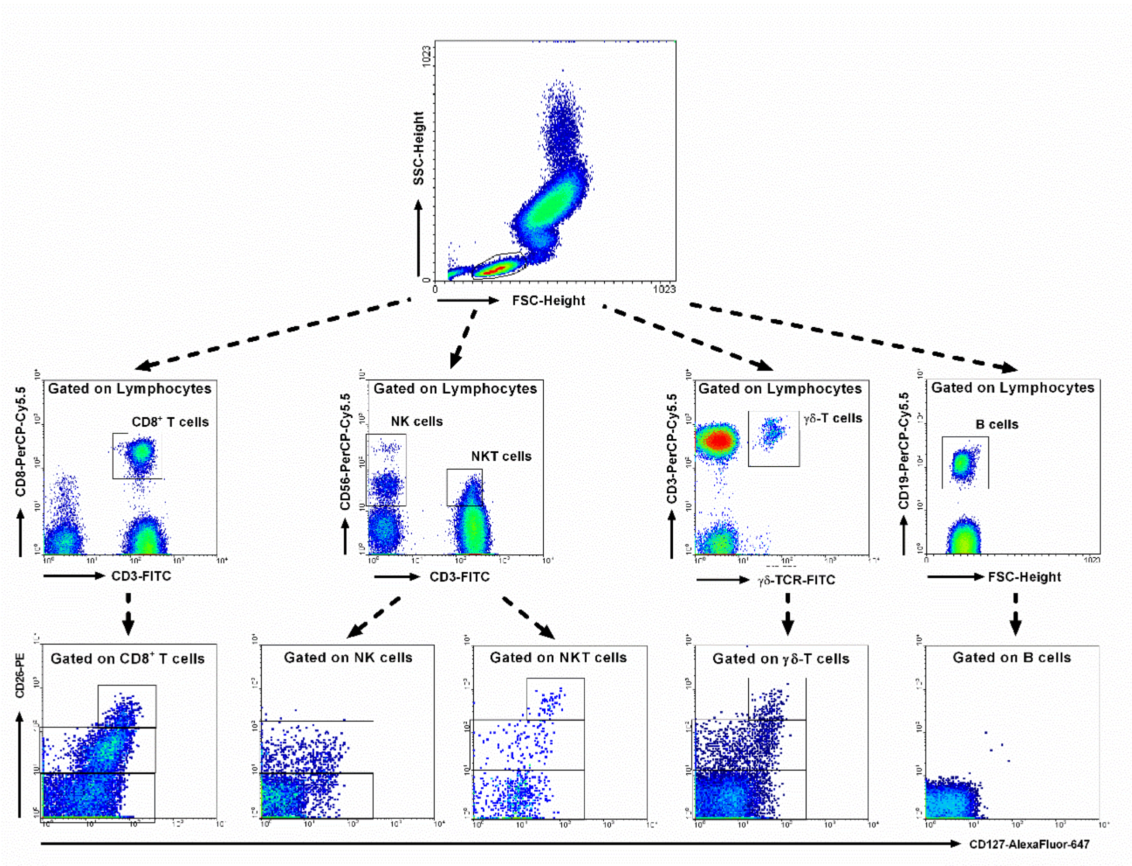

Supplementary Figure S4. Gating strategy for CD8^{+} Tc, NK, NKT, γδ-T, and B lymphocytes. After gating, expression of CD127 and CD26 was used to identify different subsets (lower row of dot-plots). CD3^{+} lymphocytes (Tc, NKT, γδ-T) were characterized by the presence of three subsets (CD26$^{-/low}$, CD26^{int}, and CD26^{high}), while CD3^{-} lymphocytes displayed a double (CD26$^{-/low}$, CD26^{int}; NK cells) or a single (CD26^{-}: B cells) phenotype.

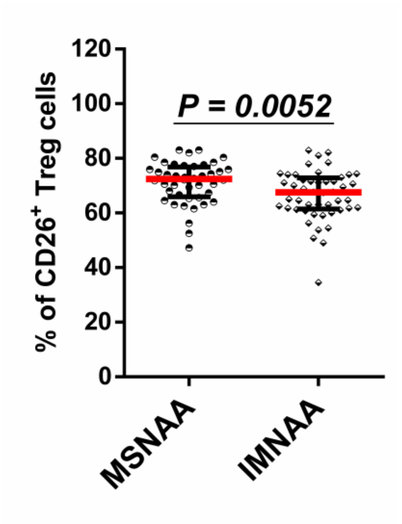

Supplementary Figure S5. Moderate-severe non-allergic asthmatics display higher percentage of CD26^{+} regulatory T cells than intermittent-mild non-allergic asthmatics. Lymphocytes from either moderate-severe non-allergic (MSNAA; N = 90) or intermittent-mild non-allergic (IMNAA; N = 102) asthmatics were gated on FSC/SSC plots and further subdivided into CD4^{-} and CD4^{+} cells. Then, regulatory T cells (Treg) were identified amongst CD4^{+} T lymphocytes according to CD25 and CD127 markers. Afterwards, the percentage of CD26^{+} cells within the Treg compartment was measured. Statistically significant difference between the different groups of donors is indicated (*P*-value).

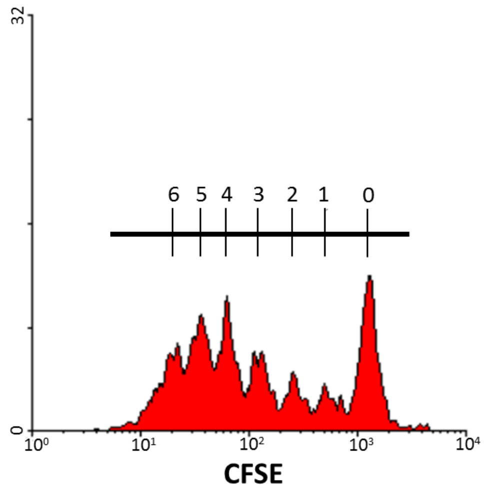

Supplementary Figure S6. Analysis of the number of cell divisions by flow cytometry. PBMCs were stained with 5 µM CFSE and cultured at 0.25 x 10^6 cells/mL in complete medium (RPMI 1640, 10% FBS, 100 µg/mL streptomycin, 100 U/mL penicillin) supplemented with 1 µg/ml PHA as a T-cell specific polyclonal stimulus. Upon 6 days of *in vitro* culture, CFSE fluorescence decay was measured by flow cytometry and allowed the calculation of the number of cell divisions. A representative histogram plot is shown, where numbers 1-6 represent the corresponding round of division, and 0 those lymphocytes that remained at a resting state. Responder frequency (Rf) was calculated as the percentage of cells that divided at least once (i.e., percentage of cells that divide from 1 to 6 times).
